# A Meta-Analysis of the Human Gut Mycobiome Using Internal Transcribed Spacer Data

**DOI:** 10.3390/microorganisms12122567

**Published:** 2024-12-13

**Authors:** Zeming Zhang, Yining Zhang, Qixiang Yuan, Zuoyi Wang, Songnian Hu, Peng Yin, Zilong He

**Affiliations:** 1 School of Engineering Medicine, Beihang University, Rd37, Xueyuan, Haidian, Beijing 100191, China; zzzzzzm@buaa.edu.cn (Z.Z.); zhangyiningscu@163.com (Y.Z.); 23101077@buaa.edu.cn (Q.Y.); wzy1048@buaa.edu.cn (Z.W.); 2 Key Laboratory of Big Data-Based Precision Medicine, Beihang University, Ministry of Industry and Information Technology of the People’s Republic of China, Beijing 100191, China; 3 State Key Laboratory of Microbial Resources, Institute of Microbiology, Chinese Academy of Sciences, Beijing 100045, China; husn@im.ac.cn

**Keywords:** gut mycobiome, ITS sequencing, meta-analysis, taxonomy classification, machine learning

## Abstract

The intestinal mycobiome is closely related to human health. There have been several reports investigating the association between the gut fungi and disease, but there is still a lack of overall assessment of the human gut mycobiome. Here, we performed a meta-analysis based on 2372 ITS (Internal Transcribed Spacer) data collected publicly online. We found that the mycobiome diversity of human gut fungi varies significantly across diseases by using EasyAmplicon, and these fungi are mainly composed of three genera, *Saccharomyces*, *Candida*, and *Aspergillus*. In addition, we performed the construction of disease prediction models based on ITS data by using the random forest model and verified the generalization ability of the models. We hope that our results will provide strong support for subsequent studies of the intestinal mycobiome.

## 1. Introduction

Mycology, the science of fungi and their interactions with the environment, has made remarkable progress in recent years in revealing the complex relationship between fungi and human health [1]. Fungi not only play key roles in nature, but are also closely linked to human health, especially when they act as pathogens causing disease [2]. For example, invasive fungal infections, such as Aspergillosis and Cryptococcosis, have become a major global public health challenge, especially in populations with compromised immune systems, and these infections often require prolonged treatment regimes, increasing healthcare burdens worldwide. In addition, symbiotic relationships between fungi and plants, such as mycorrhizal fungi (mycorrhizal fungi), are not only critical for nutrient cycling in ecosystems, but also have a profound impact on plant growth and survival [3]. These symbiotic relationships reveal the interactions between species in the microbial world that have transformed our understanding of biology, particularly in terms of species interdependence and evolution. The intestinal mycobiome is a group of fungi that live in the host gut and are an important part of the intestinal microbial community [4], with typical fungi such as *Candida albicans* and *Saccharomyces cerevisiae* [5,6]. Gut fungi are closely related to human health. They are involved in the pathology of many gut-related diseases and are strongly implicated in many immunological disorders (e.g., multiple sclerosis and alcohol use disorders) [7,8]. Given the increasing recognition of the human microbiome as a determinant of health and disease, fungi are poised to be one of central players in the integrated microbiome landscape. Therefore, research on intestinal fungi is essential.

Nowadays, there have been several studies on the intestinal mycobiome and disease. D. Iliev et al. showed that intestinal fungi act on the immune system via the innate immune receptor Dectin-1, and that alterations in this receptor may be induced by changes in the fungal community, thereby increasing the incidence of colitis [9]. Bacher et al. found that *Candida albicans* is a unique inducer of Th17 cells in pathological immune responses in the small intestine [10]. Sonja et al. found that feces from patients with alcohol hepatitis and alcohol use disorders were enriched with *Candida albicans* compared to controls by ITS sequencing [11]. However, there is still a lack of assessment of the overall impact of the gut mycobiome on human disease.

In this study, we conducted a meta-analysis on the effects of gut fungi and diseases based on online public ITS data. We screened seven studies from 87 publications, including 2372 samples, of which 1912 were in the control group and 460 were in the disease group. The results of this study deepen the understanding of gut fungi for a number of diseases. We hope that this study will further reveal the relationship between intestinal fungi and human health, and lay the foundation for research on the intestinal fungal mechanisms of some diseases. By advancing our understanding of the mycobiome, we can better elucidate the multifaceted interactions within the gut microbiota ecosystem, ultimately unlocking new pathways for precision medicine and personalized healthcare approaches.

## 2. Method

### 2.1. Public Internal Transcribed Spacer (ITS) Sequencing Data Screening and Preprocessing

We used the keyword “human gut Internal Transcribed Spacer” to search for relevant literature in PubMed, Embase, Web of Science, and other databases. Afterward, we screened the retrieved literature (Figure S1) with the following conditions: (1) the sample data were ITS sequencing data from the human gut; (2) the total sample size of each study was not less than 40 cases; (3) except for one large health cohort, the other studies included sequencing data from both disease and healthy control groups; (4) the sequencing data had been published and were available for download; (5) the metadata of the sequencing data was available. In the end, we retained data from seven studies with a total of 2372 cases [11,12,13,14,15,16,17] (Table S1). Prior to the analysis, all datasets were cross-referenced with existing sequencing repositories to eliminate duplication and ensure data integrity. Furthermore, each dataset was assessed for completeness and filtered to exclude any samples lacking comprehensive metadata, ensuring a robust analysis foundation.

### 2.2. Internal Transcribed Spacer (ITS) Data Processing and Taxonomy Classification

Raw sequencing data were downloaded from the NCBI database (https://www.ncbi.nlm.nih.gov/sra, accessed on 19 March 2024) or the European ENA database (https://www.ebi.ac.uk/, accessed on 19 March 2024). Raw data were preprocessed using trim_galore to remove adapter sequences and low-quality bases. Quality control included the use of chimera filtering to remove potential sequencing artifacts, ensuring only high-confidence reads were classified. Post-taxonomic assignment and normalization procedures were applied to manage sequencing depth variability across samples, contributing to the reliability of diversity indices calculations. We used QIIME2 (version 2023.9, QIIME 2 Development Team, Flagstaff, AZ for USA) to analyze the data [18]. Taxonomy classification was performed according to UNITE (version 18.07.2023, University of Tartu, Tartu, Estonia) database. For subsequent analyses, we retained a genus-level relative abundance profiling. We assessed the relationship between fungal composition and respective diseases in each subgroup by the Bray–Curtis distance, to visualize the distribution of samples based on pairwise distances. The principal coordinate analysis (PCoA) was generated using the first two principal coordinates. We reflected the abundance and diversity of the microbial community through the Evenness (an index that reflects whether the species in an ecosystem are evenly distributed or whether they are dominated by a small number of species) and Shannon (an important indicator used to measure the diversity of species in an ecosystem) indices. All of the above analyses were implemented using the EasyAmplicon (version 1.21, Yongxin Liu Team, China) platform [19].

### 2.3. Internal Transcribed Spacer (ITS) Abundance Differential Microbes Analysis

We analyzed the differential microbiota between disease and control groups at the genus level in each of the seven datasets using LEfSe (an analytical tool for interpreting biomarkers, version 1.1.2, Huttenhower Lab, Boston, MA, USA). This analysis first used a non-parametric Kruskal–Wallis rank sum to detect species with significant differences in abundance between different groups to find taxa with significant differences in abundance, and then the linear regression analysis (LDA) was used to estimate the magnitude of the effect of abundance to find species with significant differences (LDA > 2). A permutation test, consisting of 1000 permutations, was included to confirm the robustness of findings, providing a rigorous assessment of the biological relevance of the potential biomarker taxa identified. The above analyses were implemented through the LEfSe component provided by the imageGP platform (accessed on 21 Jay 2024, Yongxin Liu Team, China).

### 2.4. Disease Prediction Model Construction Based on Internal Transcribed Spacer (ITS) Data

We used seven different disease-control datasets from seven studies (type 1 diabetes mellitus, T1DM; alcoholic hepatitis, AH; alcohol utilization disorder, AUD; non-alcoholic fatty liver disease, NAFLD; depressionDPR; rheumatoid arthritis, RA; multiple sclerosis, MS) for modeling and standardized each dataset to ensure that the eigenvalues were within the same range of magnitude. We also dealt with missing values and outliers to ensure data quality and consistency. Model construction was performed using the randomForest function from the randomForest package. Parameter settings included the tree (number of decision trees, set to 1000), using the 10-fold cross-validation, repeated five times, and selecting the best mtry (a parameter for randomized feature selection that controls the number of features that can be selected for each tree when splitting nodes) model for training and testing. Seven random forest models were trained separately using seven different disease datasets. On each trained model, the same disease dataset was used for testing. For each random forest model, we calculated the predictive probability using the test dataset. The ROC curves were calculated using the roc function in the pROC package (version 1.18.5) to obtain the FPR (False Positive Rate) and TPR (True Positive Rate) values and the AUC values for each model were calculated using the auc function in the pROC package. Moreover, to assess the generalizability of the models, the 10-fold cross-validation was combined with stratified sampling to maintain the proportion of cases and controls within each fold, guarding against overfitting and promoting model applicability to unseen data. Similarly, we also tested each trained model with different disease datasets to obtain a table of cross-validation results where the value of each cell represents the AUC value of the model trained using a specific disease dataset on other disease datasets and the heatmap table.

### 2.5. Data Statistics and Visualization

All processed data, if not otherwise stated, were loaded in the R language environment (version 4.3.2), and analyzed and visualized. Comparisons between the two groups were made using the Wilcox test, and correlation analyses were performed using the Spearman correlation test. All tests of significance were two-sided, with *p*-values < 0.05 (two-group comparisons) or corrected *p*-values < 0.05 (multiple-group comparisons) considered statistically significant. Graphical outputs were refined to include confidence intervals and statistical test annotations, enhancing interpretability. Visualization strategies such as multi-panel plots and interactive dashboards were utilized to portray complex dataset relationships effectively, facilitating exploratory data analysis and hypothesis generation.

## 3. Result

### 3.1. Data Screening and Quality Control

In total, we collected ITS data from seven studies. After quality control, we excluded samples with less than 5000 reads and obtained a total of 2372 ITS samples (Table 1), including 1912 cases in the control group, which contained a cohort of 1633 healthy older adults, and 460 cases in the disease group. The disease group mainly contained MS, AUD, T1DM, AH, DPR, RA, and NAFLD, with an average sample size of 105 cases per group, and higher sample sizes in the DPR group (*n* = 255) and the T1DM group (*n* = 155), for a total data volume of 24.12 G.

### 3.2. α-Diversity and β-Diversity Analysis of Intestinal Mycobiome

**Figure 1:**
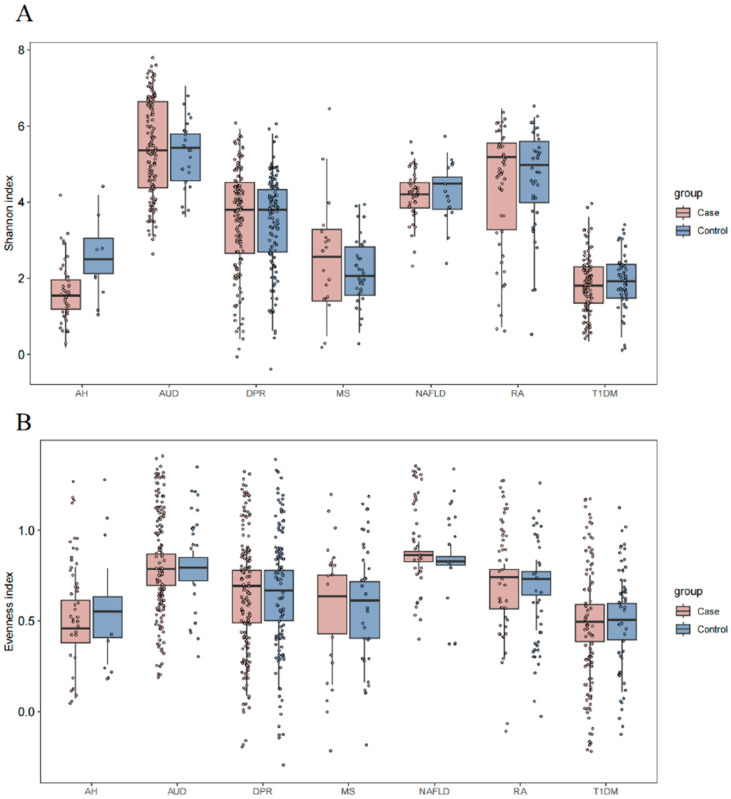
(**A**) The distribution of the Shannon index of human gut mycobiome. (**B**) The distribution of the Evenness index of human gut mycobiome.

**Figure 2:**
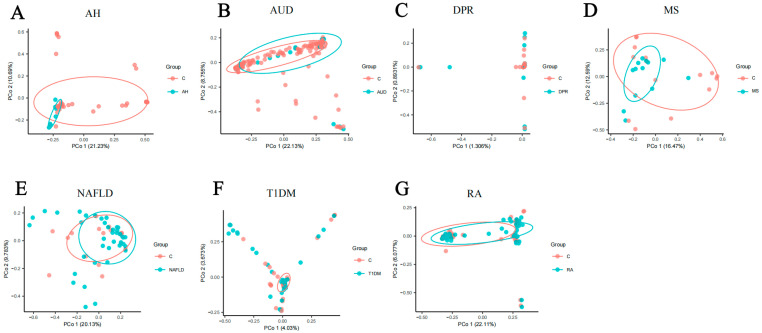
The PCoA analysis of human gut mycobiome.

We assessed the α-diversity of gut mycobiome under different disease models based on the Shannon and Evenness indices (Figure 1). From the Shannon index, the overall AUD group had the highest median, followed by the RA group, while the AH and T1DM groups had lower medians. In terms of the Evenness index, the median was higher in the NAFLD and AUD groups, and the Evenness index was relatively concentrated in the NAFLD group, while the median was lower in the AH and T1DM groups. As a result of the above analyses in the seven groups, the differences between the disease and control groups were not significant (*p* > 0.05) for both indices for each disease studied. At the same time, we also assessed the β-diversity of the gut mycobiome under different disease models (Figure 2). From the results of PCoA analyses, the samples of intestinal microbiota in the seven diseases were not significantly separated. Most of the samples of the disease group overlapped with those of the control group, while the distribution of sample points in the control group was more dispersed.

### 3.3. Taxonomy Classification of Intestinal Fungi

**Figure 3:**
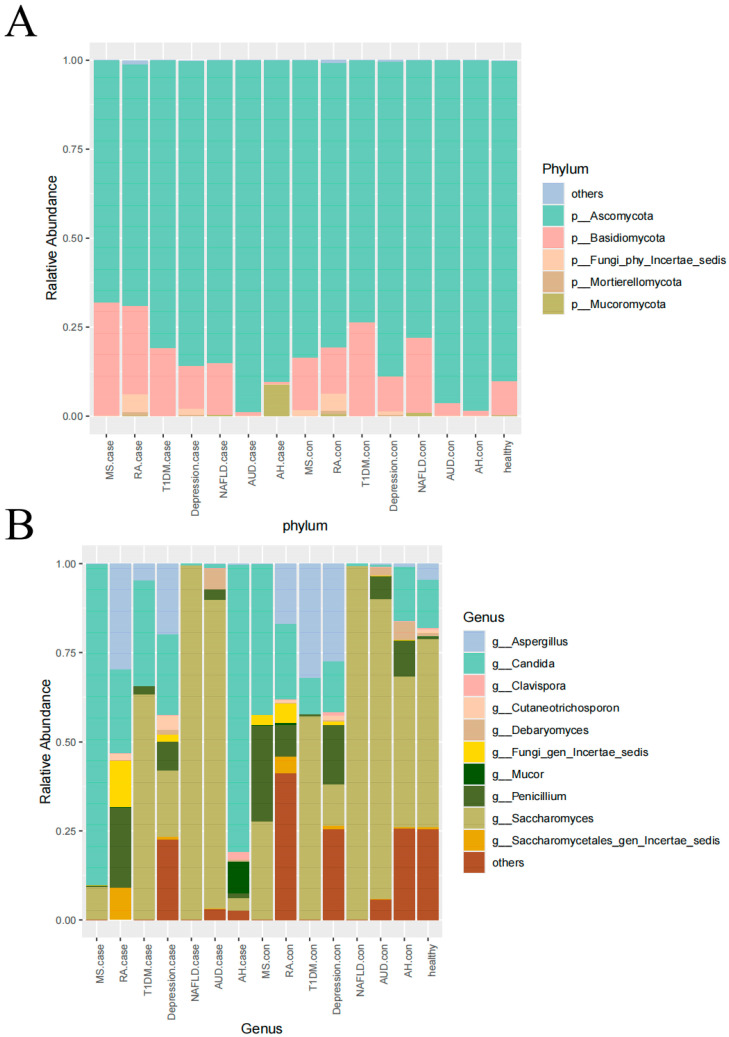
(**A**) Taxonomy classification of human gut mycobiome at the phylum level. (**B**) Taxonomy classification of human gut mycobiome at the genus level.

We further analyzed the intestinal fungal composition of disease versus control samples at the phylum level and genus level in each group (Figure 3). At the phylum level, the groups were mainly focused on Ascomycota and Basidiomycota, with Ascomycota having the highest abundance and the first abundance among disease versus control samples in multiple groups; Basidiomycota ranked second in abundance among multiple groups. In addition, other phyla with high abundance contained Mucoromycota and the Fungi_phy_Incertae_sedis. At the genus level, the groups were dominated by the genera *Candida*, *Saccharomyces*, and *Aspergillus*, which occupied the top three abundance in several groups, both in disease and control samples. Other genera with relatively high abundance included *Debaryomyces*, *Penicillium*, and *Mucor*. What is more, for genus-level gut fungi in healthy individuals, we performed core microbiota identification (Relative Abundance > 0.01 and Prevalencerate > 0.5). Among the seven control samples, the core microbiota in three or more groups were *Saccharomyces*, *Candida*, *Aspergillus*, and *Penicillium*. In the cohort of 1633 healthy individuals, the core microbiota were *Saccharomyces*, *Candida*, and *Aspergillus* (Table S2).

### 3.4. Identify Abundance-Differentiated Intestinal Mycobiome Between Disease and Control Groups

**Figure 4:**
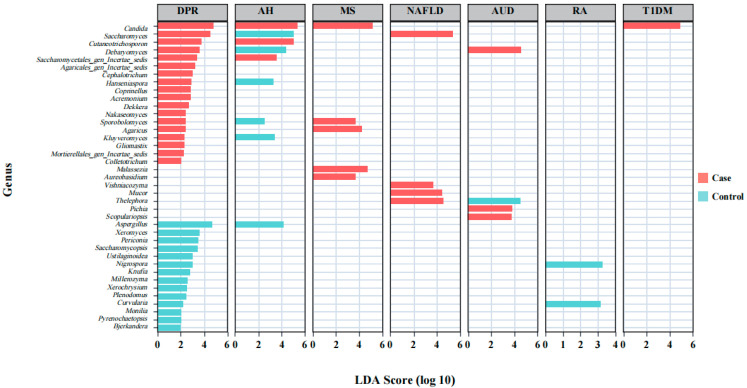
LEfSe analysis of human gut mycobiome at the genus level.

We analyzed the abundance differential microbiota at the genus level for seven different diseases (Figure 4). The results showed that in each disease, we were able to find its major differential fungi, with some groups having more, such as the AH group and DPR, while some groups having fewer ones, such as the T1DM group and NAFLD group. In the analysis of the AH group, there were three abundance differential fungi in the disease group including *Candida* and *Saccharomycetales_gen_Incertae_sedis* (LDA Score > 3.5). There were six abundance differential fungi (LDA Score > 2) in the control group, including *Saccharomyces* and *Aspergillus.* In the AUD group, there were three major abundance differential fungi in the disease group (*Debaryomyces*: LDA Score > 4.2, *Pichia*: LDA Score > 3.5, *Scopulariopsis*: LDA Score > 3.5). In the control group, there was one main fungus, *Thelephora* (LDA Score > 4.2). In the DPR group, there were 18 abundance differential fungi in the disease group represented by *Candida* and *Saccharomyces* (LDA Score > 5.0). As for the control group, it had 14 abundance differential fungi in which *Aspergillus*, *Saccharomycopsis*, and *Xeromyces* had greater LDA values (LDA Score > 3.4). Analyzing the disease groups in MS, *Candida*, *Sporobolomyces*, *Malassezia*, *Agaricus,* and *Aureobasidium* were considered to be differentiated (LDA Score > 3.6). In the NAFLD group, there were four major abundance differential fungi (LDA Score > 3.6), *Mucor*, *Thelephora*, *Saccharomyces,* and *Vishniacozyma*. In the RA group, there were two abundance differential fungi (LDA Score > 2.0) in the control group, including *Nigrospora* and *Curvularia*. In the T1DM group, we found only one abundance differential fungus in the disease group, *Candida*, whose LDA Score was close to 5.

### 3.5. Disease Prediction Modeling Based on Intestinal Internal Transcribed Spacer (ITS) Data

**Figure 5:**
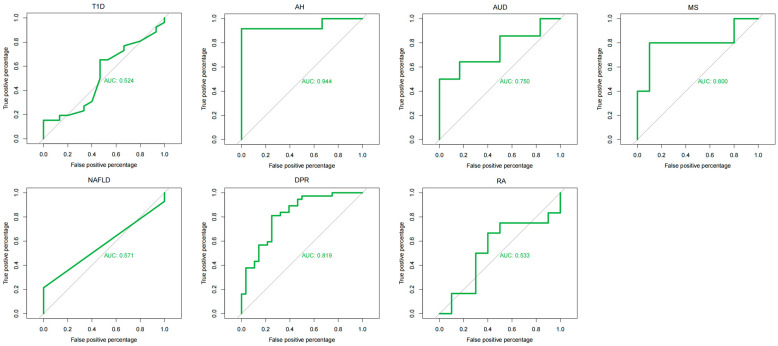
Random forest model for seven studies based on ITS data.

**Figure 6:**
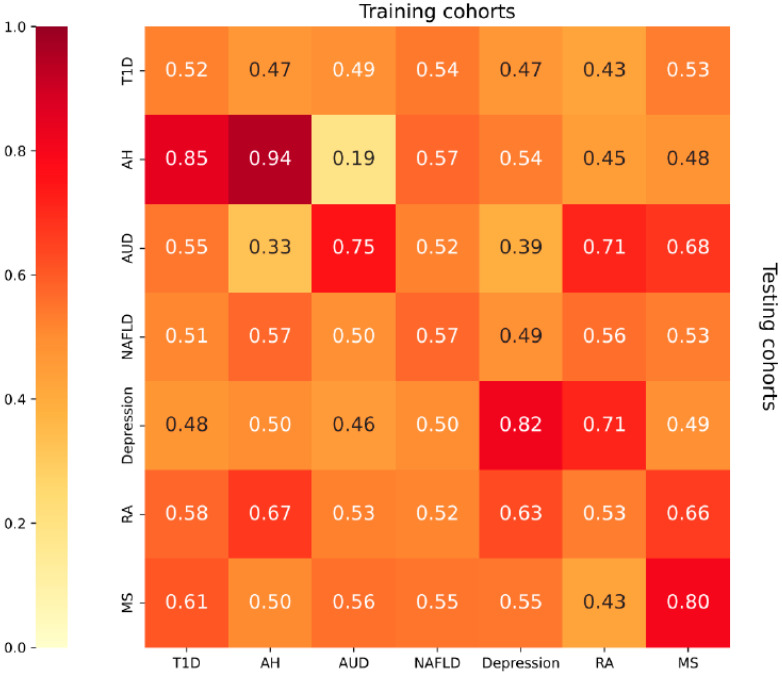
The heatmap of prediction modeling based on intestinal ITS data.

In this study, we trained random forest models for seven studies and plotted their ROC curves (Figure 5) to assess the classification performance of the models. From the AUC values, the AH group has the highest AUC value of 0.944, which is the best prediction. The MS and DPR groups have better performance with AUC values of 0.800 and 0.819, respectively. The AUD group has an AUC value of 0.750. However, the AUC values of the NAFLD group, the RA group, and the T1DM group are 0.571, 0.533, and 0.524, which are lower than 0.6, indicating the poor classification performance of these models. Furthermore, we also evaluated the generalization ability of the random forest model by a cross-validation method using datasets from seven different diseases (Figure 6). According to Figure 6, we found that there are some models with high generalization ability, such as the DPR group model which showed better cross-disease generalization ability, with its AUC value of 0.711 on the RA group, respectively. The AH group model showed a strong cross-disease generalization ability with an AUC value of 0.846 on the T1DM group. However, there are also some models with differences in the generalization ability on different test sets. Some models with weak generalization ability, e.g., the model trained on the AH group data has an AUC value of 0.846 on the T1DM group data, but only 0.190 on the AUD group data. The performance of the AUD group fluctuates to a great extent on different test sets, in particular on the NAFLD group and the DPR group, with an AUC value of 0.521 and 0.389.

## 4. Discussion

Currently, ITS sequencing is the main approach to studying human mycobiome, and the commonly used amplification regions are ITS1 and ITS2 [20], etc. Lai et al. evaluated a large number of ITS1 and ITS2 intestinal fungal samples and found that the intestinal mycobiome was classified into three enterotypes [21]. Shuai et al. evaluated the determinants and stability of the gut fungal microbiota of more than 1000 middle-aged and elderly people based on ITS sequencing [17]. However, there is still a lack of systematic assessment of the human gut mycobiome and disease, especially whether it can be used as a reliable molecular marker for disease prediction, which remains to be investigated. In this study, we systematically assessed the differences between healthy and diseased patients with intestinal fungi in multiple cohorts based on publicly available ITS data online. We constructed a disease prediction model as well as generalization properties, which provide an important theoretical basis for the systematic understanding of the link between the intestinal mycobiome and disease. This study initially reveals the possibility of ITS as a molecular marker for disease prediction.

The intestinal microbial community is complex and consists mainly of microorganisms such as bacteria, fungi, as well as viruses [22,23]. Currently, gut microbiology is mainly focused on the association between changes in bacterial communities and host health, while the effect of fungal diversity on the host has not been systematically revealed. In our study, we systematically compared gut fungal diversity in seven diseases and found significant changes in fungal diversity across diseases, suggesting that changes in gut fungi are closely related to host health. In terms of microbiological composition, the three most common genera of intestinal fungi were *Saccharomyces*, *Candida*, and *Aspergillus*, and were frequently identified in the differential flora of different diseases, suggesting that these are the three most important species of intestinal fungi. *Saccharomyces* is a genus of yeasts, most commonly *Saccharomyces cerevisiae* [24] and some species such as *Saccharomyces boulardii* [25] are used as probiotics. The *Saccharomyces* genus of gut microorganisms plays an important role in human health and disease [26], and not only does it help to maintain intestinal health [27], but it may also play a role in the treatment of certain diseases [28]. *Candida* is also a common genus of fungi, the best-known member of which is *Candida albicans* [29]. Candida fungi also play a role in the gut microbiota, such as interacting with host cells, influencing host cell growth and signaling pathways [30,31], affecting metabolic health and regulating immune responses, etc. The role of *Candida* fungi in the gut microbiota may vary depending on individual differences, dietary habits, health status, etc. *Aspergillus* is mainly distributed in the natural environment as well as on the surface of the human body [32]. It mainly causes infections in the host, but it is less studied in the gut, and it is speculated that *Aspergillus* may be involved in metabolism, immunomodulation, etc. [33]. The main role it plays in the gut needs to be further revealed. By analyzing the intestinal ITS data, we initially identified the core fungal genera in the intestine, which lays a theoretical foundation for further research in the future.

Gut microbes have now proved their value as molecular markers in a variety of disease prediction models [34]. Diabetes [35], enteritis [36], and cardiovascular disease have all been modeled by differential gut microbiota with excellent performance [37]. With the improvement of sensitivity as well as accuracy, this non-invasive molecular marker will gradually replace blood as well as other tissue samples as a new routine assay [38]. However, most of the molecular markers for gut microbes are currently based mainly on bacteria in gut microbes, mainly because bacteria are abundant and diverse in gut microbes, but fungi, which are also members of gut microbes, have the potential to be molecular markers for disease modeling that has not been evaluated. In our study, for the first time, we systematically assessed the modeling performance of ITS data in different diseases and their ability to generalize. We found that the excellent performance demonstrated by gut fungi in certain diseases such as AH and T1DM, and the generalization ability regardless of the model is also noteworthy. Overall, gut fungi have the potential to become molecular markers and perform well in the prediction of specific diseases, which is worth expanding the sample size for an in-depth study in the later stage.

There are still some limitations in this study. Firstly, although this study contains enough sample data as a whole, the paired data of case-control are relatively small, especially for the case of a single disease, and we should continue to increase the samples at a later stage to detect the fungi biomarkers as well as the stability of the model. Secondly, the current study of fungal groups is still mainly based on amplicon technology, which can capture fungal sequences well, but due to its technical limitations, the annotation of the species level is not very accurate, and metagenome sequencing for the quantification of the fungal group is also very limited, which may be mainly due to the current intestinal fungi reference genome being a relatively small number, and later needs to increase the genomic sequences of the fungal single strains. In addition, the ITS data do not reflect well the function and diversity of genes within a single fungal species, and research on the impact of fungal function on disease needs to be strengthened. Finally, the advantages of ITS as a molecular marker for disease prediction compared to bacteria need to be further demonstrated.

## 5. Conclusions

We conducted a meta-analysis of seven diseases by integrating online public ITS data. We found that the diversity of intestinal fungi varied greatly among different diseases. The intestinal fungi were mainly composed of three genera, *Saccharomyces*, *Candida*, and *Aspergillus*, and these three genera appeared in the different diseases. Constructing a disease prediction model based on the ITS data, we found that the ITS data have a relatively good performance for specific diseases (e.g., AH and T1DM), and there is a certain generalization ability. The multi-level integrated analysis in combination with other histological data (e.g., macrogenomic, metabolomic) may provide richer information on the potential mechanisms of intestinal fungi in disease. In summary, we hope that our findings will provide important clues for subsequent studies of intestinal fungi.

## Figures and Tables

**Table 1:** Summary of ITS data on the available database.

Cohort Description	PMID	Disease	Number of Disease Group Samples	Number of Control Group Samples	Total
Intestinal fungal dysbiosis and systemic immune response to fungi in patients with alcoholic hepatitis	31228214	AH	36	9	45
Altered gut bacterial–fungal interkingdom networks in children and adolescents with depression	37003434	DPR	145	110	255
Multiple sclerosis patients have an altered gut mycobiome and increased fungal to bacterial richness	35472144	MS	17	27	44
The fecal mycobiome in non-alcoholic fatty liver disease	34896404	NAFLD	41	16	57
Alterations of gut fungal microbiota in patients with rheumatoid arthritis	35251791	RA	47	39	86
Women with type 1 diabetes exhibit a progressive increase in gut *Saccharomyces cerevisiae* in pregnancy associated with evidence of gut inflammation	35051423	T1DM	99	56	155
Mapping the human gut mycobiome in middle-aged and elderly adults; multiomics insights and implications for host metabolic health	35017200	Healthy	0	1633	1633
Intestinal fungal dysbiosis and systemic immune response to fungi in patients with alcoholic hepatitis, the fecal mycobiome in non-alcoholic fatty liver disease	31228214	AUD	75	22	97
Total			460	1912	2372

## Data Availability

The data presented in this study are openly available in PubMed with the following DOIs and reference numbers: 10.1002/hep.30832 [8], 10.1016/j.jad.2023.03.086 [9], 10.1371/journal.pone.0264556 [10], 10.1016/j.jhep.2021.11.029 [11], 10.7717/peerj.13037 [12], 10.1016/j.diabres.2022.109189 [13], 10.1136/gutjnl-2021-326298 [14].

## Supplementary Materials

The following supporting information can be downloaded at: https://www.mdpi.com/article/10.3390/microorganisms12122567/s1, Figure S1. ITS data screening process. Table S1. The metadata of ITS samples. Table S2. The abundance and frequency of ITS data.
