# Supplementary figures and images for: A Meta-Analysis of the Human Gut Mycobiome Using Internal Transcribed Spacer Data
Source: Microorganisms. 2024 Dec 13;12(12):2567. doi: 10.3390/microorganisms12122567 (PMC11678510; doi:10.3390/microorganisms12122567)

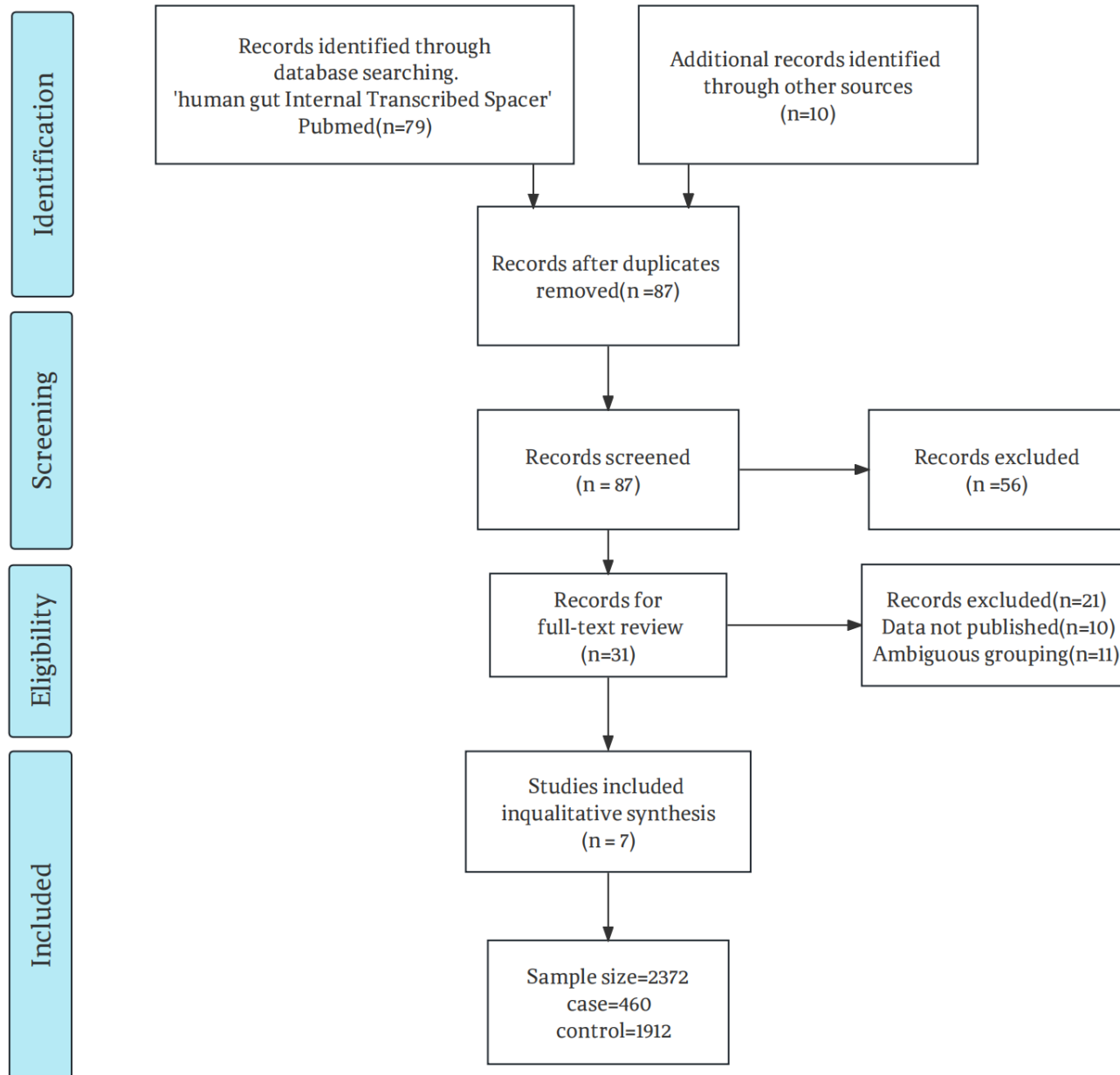

Supplement: Supplementary file 1 [file microorganisms-12-02567-s001.zip › Figure S1.pdf]

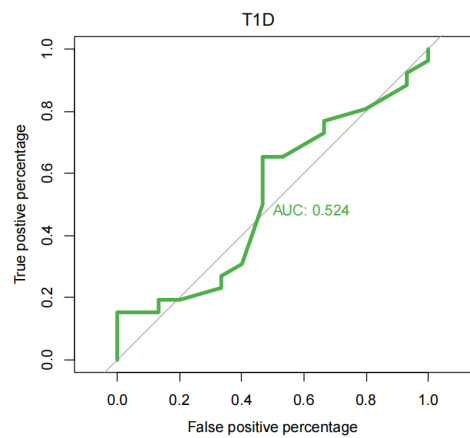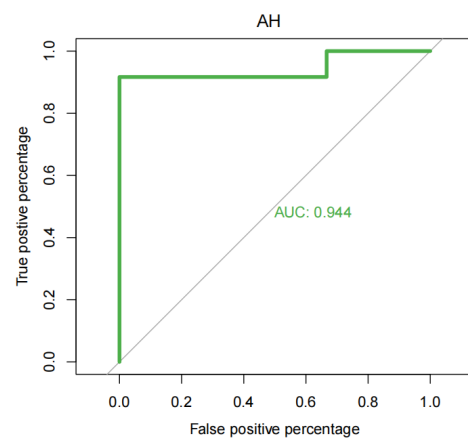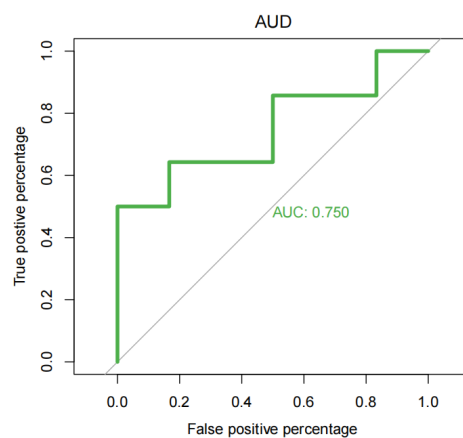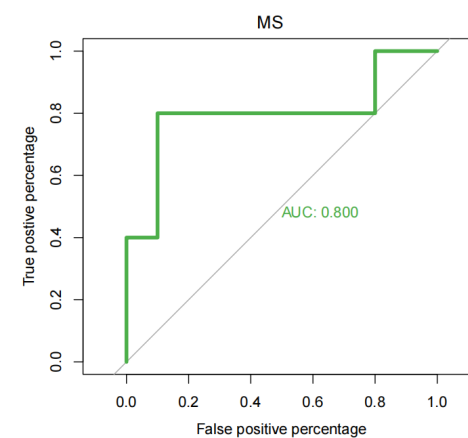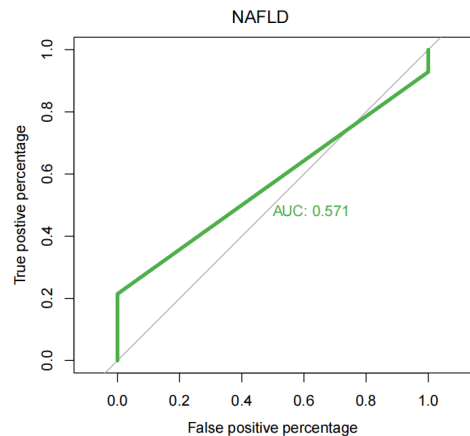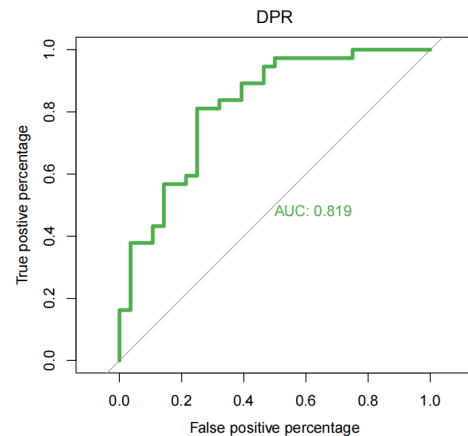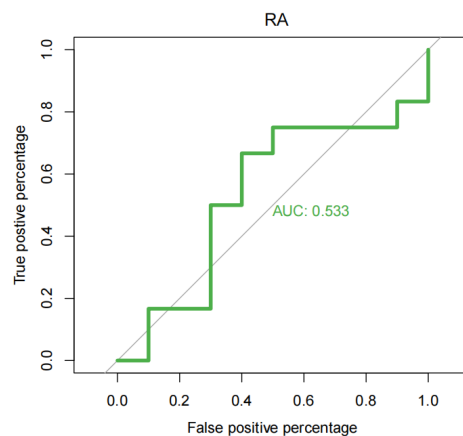

Supplement: Supplementary file 1 [file microorganisms-12-02567-s001.zip › Figure S2.pdf]
